# Effectiveness of Physical Activity Interventions on Acute Inpatient Mental Health Units on Health Outcomes: A Systematic Review

**DOI:** 10.1111/inm.70017

**Published:** 2025-02-23

**Authors:** Michael Graham, Philip Hodgson, Laura Fleming, Alison Innerd, Nicola Clibbens, Wendy Hope, Luke Aston, Michelle Glascott

**Affiliations:** ^1^ School of Health and Life Sciences Teesside University Middlesbrough UK; ^2^ Tees, Esk and Wear Valleys NHS Foundation Trust Darlington UK; ^3^ York St. John University York UK; ^4^ Department of Nursing and Midwifery Northumbria University Newcastle‐upon‐Tyne UK; ^5^ Cumbria, Northumberland, Tyne & Wear NHS Foundation Trust Newcastle‐upon‐Tyne UK; ^6^ Cumbria, Northumberland, Tyne & Wear NHS Involvement Bank St. Nicholas Hospital Newcastle‐upon‐Tyne UK; ^7^ Tees Esk and Wear Valleys NHS Foundation Trust ARCH Recovery College Durham UK

**Keywords:** AMHU's, mental health, SMI

## Abstract

Physical activity has been shown to improve outcomes across a range of physical and mental health conditions as an adjunct or standalone intervention for many mental disorders. The outcome and effectiveness of physical activity in acute mental health units are less well understood. Systematic searches were completed in three databases (CINAHL, MEDLINE, and PsycINFO). Eligible studies were published between March 2013 and February 2024, included a physical activity intervention for inpatients on acute mental health units, and reported primary quantitative, qualitative, or mixed methods data for patients between 18 and 65 years of age. Participants must have had a primary diagnosis of a mental health condition with or without physical comorbidities. Data extracted included reported components of the interventions and individual health outcomes. Methodological quality and risk of bias was assessed using the mixed methods appraisal tool and cochrane risk of bias tools for randomised and non‐randomised controlled trials. Twelve studies were identified for review (combined sample size of 560). Seven studies reported improvements in mental health outcomes, and two reported improvements in physical health outcomes in favour of the intervention group. There was a large variation between intervention characteristics and clarity in reporting. Assessment and measurement of outcomes contributed to a high risk of bias among included studies due largely to self‐assessment. Physical activity interventions on AMHUs have the potential to contribute to improvements in mental and physical wellbeing beyond that experienced from usual treatment practices (e.g., medication). However, further work is needed in the specific context of acute mental health units regarding the development and evaluation of physical activity interventions.

## 1 Introduction

In the UK and worldwide, there has been an increased emphasis to align the level of care and urgency delivered for physical health conditions to that of mental health conditions (Royal College of Psychiatrists 2013; European Commission 2023). Determinants of health, such as physical activity levels and the avoidance of harmful behaviours, such as drug and alcohol abuse, can have a significant impact on good physical and mental health.

Interventions including physical activity have been shown to improve outcomes across a range of physical health conditions (Kandola and Osborn 2021) including cardiovascular disease, diabetes, cancer, and common mental health disorders (Wilmot et al. 2012; Kyu et al. 2016; Bennett et al. 2017; Schuch et al. 2018, 2019). Translation of these interventions into health care delivery has shown some success for physical health (Husk et al. 2020) but implementation in mental health care has been slower (Way et al. 2018; National Institute for Health and Care Excellence 2022).

Physical activity is recommended as an adjunct or standalone intervention across a range of mental health conditions, including depression, anxiety disorder, psychoses, and substance use (Ashdown‐Franks et al. 2020; Lovell et al. 2015). Studies have shown physical activity to alleviate symptoms of depression (Rebar et al. 2015), anxiety disorders (Jayakody et al. 2014), bipolar disorder (Sylvia et al. 2010), and schizophrenia and psychoses (Vera‐Garcia et al. 2015; Firth et al. 2015; Firth et al. 2017; Keller‐Varady, 2018). Physical activity has also been shown to be effective as an adjunct to standard treatment for substance misuse (Dolezal et al. 2013; Giesen et al. 2015). Symptom improvements, including sleep (Lederman et al. 2019) and reduced suicidal ideation (Kvam et al. 2016), have also been observed.

People with serious mental health conditions (including schizophrenia and bi‐polar disorder) are at increased risk of early mortality by between 10 and 20 years compared to age‐matched controls (Laursen et al. 2014; Plana‐Ripoll et al. 2020), and this gap is widening (Hayes et al. 2017). Whilst suicide accounts for a proportion of early mortality in the population with serious mental illness, a complex aetiology related to co‐morbid physical and mental health conditions, alongside cardiometabolic side effects from prescribed medicines, accounts for approximately 70% of early deaths (Vancampfort 2015).

Acute mental health units (AMHU's) provide care for people experiencing an acute and severe episode of a mental illness, where risks to self and others prevent community‐based interventions (McAllister et al. 2021). A large proportion of people on AMHU's are detained under conditions of the Mental Health Act (Care Quality Commission 2022) or international equivalents (Cronin et al. 2017) restricting their freedom to leave the AMHU unless granted by the responsible clinician. Rates of detention have been shown to have risen at an alarming rate (Rains et al. 2019, 2020). This restriction of freedom and limited access to activities can contribute to a sense of boredom and frustration for individuals, increasing rates of self‐harm and aggression (Foye et al. 2020).

Given the often‐high use of psychotropic medication (Bauer et al. 2016) coupled with low health literacy in this population (McLean et al. 2023; Degan et al. 2019), physical activity is an important intervention to promote healthy behaviours, both on the ward and after discharge (Westman et al. 2019). The link between physical activity and mental wellbeing has been suggested, but to date, reviews have not focused on effects for those in AMHUs.

Implementation of physical activity interventions in AMHU's is challenged by the acute and varied nature of the mental health presentations in the population, the short stay of those admitted and the often very frenetic nature of the ward (McAllister et al. 2021). Taking these issues into account, there is a need for a systematic review to synthesise the current evidence to guide the design and implementation of future physical activity interventions that aim to improve physical and mental well‐being, reduce stress, anxiety, and levels of anger, relieve boredom, and improve the ward atmosphere with a view to reducing distressing incidents on AMHU's.

## 2 Methods

This systematic review was registered with The Open Science Framework on the 22nd of May 2023 (https://doi.org/10.17605/OSF.IO/GQRZY) and follows the updated preferred reporting items for systematic reviews and meta‐Analysis (PRISMA) statement (Page et al. 2021).

### 2.1 Search Strategy

The search strategy included search terms/keywords using the population, intervention, outcomes (PIO) approach for systematic reviews and comprised three facets relating to (1) acute inpatient mental health, (2) physical activity, and (3) mental health. Search terms were identified by the study team with expertise including lived experience, mental health, nursing, sports science, and physiotherapy. Pilot searches were conducted by an information scientist in July 2022 and changes made to the search terms accordingly. The Boolean operators AND and OR were used alongside truncation. Searches were limited to locate studies published since 2013, coinciding with the introduction of a ‘Parity of Esteem’ policy on mental health (Royal College of Psychiatrists 2013) and to identify intervention settings that more accurately reflect the current clinical landscape of AMHU's. The included studies were published in the English language. A full search term can be seen in the Supporting Information (‘search terms’).

Targeted grey literature searches were conducted in government and charity webpages and NICE evidence using truncated search terms in November 2022 and updated in January 2024. In addition, two lived experience study team members identified key documents for inclusion not identified via database searches.

#### 2.1.1 Information Sources

Initial searches were carried out by an information specialist in three bibliographic databases: CINAHL (EBSCO interface), MEDLINE (EBSCO interface), and psycINFO (Proquest interface) in October 2022. The databases were chosen to provide a broad coverage of relevant publications from medical, specialist mental health and psychiatry, and nursing and allied health professions. Updated searches were conducted on 29th January 2024. Results were limited to publications since 2021, and these were then compared with the previous results, and duplicates were removed.

### 2.2 Eligibility Criteria

Articles eligible for inclusion in this review reported primary randomised or non‐randomised quantitative, qualitative, or mixed methods data and were published between March 2013 and January 2024. Articles were included if they reported participants aged between 18 and 65 years with a primary diagnosis of a mental health condition or with physical co‐morbidities (provided the primary focus is on mental health) receiving any form of physical activity intervention of any duration or intensity, in groups or individually, delivered/received on an acute inpatient mental health ward. The physical activity intervention could be delivered by any staff member (including fitness instructors, nurses, physiotherapists, and activity co‐ordinators), and these staff may or may not have had specific training in physical exercise or sport coaching.

Within our review, AMHUs were defined as hospital wards with a primary function to treat acute episodes of mental illness over a short time (usually no longer than 3 months). Inpatients may be detained using country‐specific mental health legislation or admitted informally. Included studies reported outcome measures or reported experiences specifically related to the effect of physical activity on individual physical or mental health, with a primary focus on mental health. Studies reporting the effect of physical activity on service‐level outcomes such as the rate of incidents of violence, self‐harm or ward atmosphere were also included.

Studies were excluded if the primary focus was on physical health conditions, learning disabilities, or dementia, studies of people under the age of 18 years or over the age of 65 years, or if the intervention was delivered/received in the community or was deemed to be a non‐acute mental health setting (i.e., longer stay wards (usually more than 3 months), forensic or other specialist services or psychiatric intensive care units). In addition, studies published prior to March 2013, previous systematic reviews, cross‐sectional, and observational studies where no physical activity intervention was evaluated were excluded.

Finally, texts that were not available in the English language were excluded. A full description of the inclusion and exclusion criteria is presented in Table 1.

**TABLE 1:** Eligibility criteria and associated codes for study selection/exclusion.

Exclusion criteria	Code
Irrelevant topic	X
Research design/article type (Systematic reviews, cross sectional, conference abstracts, guidance documents, government reports)	E1
Year of publication (published prior to 2013)	E2
FT not available in English	E3
Interventions not including physical activity/no specific PA intervention	E4
Non mental health settings	E5
NON‐Acute wards/units	E6
Community settings	E7
Population under 18 or over 65, no diagnoses of mental illness	E8
Primary diagnosis of learning disability, physical health condition, substance use	E9
No physical or mental health outcomes reported	E10

### 2.3 Selection Process

Screening was carried out in two stages, first at title and abstract and second at full text. Two members of the team independently screened 100% of the returned articles at first and second selection. Discrepancies were initially discussed between the two team members, with any further discrepancies discussed and resolved with the whole team.

### 2.4 Data Collection

A data extraction instrument was created in Microsoft Excel using the template for intervention description and replication (TIDieR) checklist as a template (Hoffmann et al. 2014). Data extraction was completed by two teams, each comprising three members. The amended framework was then piloted on two papers to ensure consistent use. Eligible articles were distributed evenly between the teams, and one of the remaining study teams completed a quality control procedure to ensure that both teams had used the tool consistently by sampling 10% of the articles. Discrepancies were resolved through discussion with the whole team before compiling and synthesising data.

#### 2.4.1 Data Items

Data extracted from each study included study details, setting, patient population data, physical activity intervention type, session frequency, duration, and intensity, physical activity outcome data, physical and mental health outcomes, patient experiences, as well as staff expertise and experience.

The TIDieR framework (Hoffmann et al. 2014) was used as a template in which to produce a comprehensive descriptive and analytical presentation of the intervention details and outcomes.

### 2.5 Quality Appraisal and Risk of Bias

The Mixed Method Appraisal Tool (MMAT) (Hong et al. 2018) was used to appraise and extract data on the quality of each included study. The Cochrane Risk of Bias 2.0 (RoB2.0) tool (Sterne et al. 2019), the Risk of Bias in Non‐Randomised Studies of Interventions (ROBINS‐I) (Sterne, Hernán, et al. 2016; Sterne, Higgins, et al. 2016), and the Risk of Bias in Non‐Randomised Studies of Exposures (ROBINS‐E) (Higgins et al. 2023) were used to evaluate the risk of bias for each quantitative study. All included studies were appraised by two authors, with studies distributed evenly and a 10% sample checked for consistency. Studies were not excluded based on quality.

Studies assessed using the RoB2.0 were scored as either ‘low risk’, ‘some concerns’, or ‘high risk’ for each domain; (1) bias arising from the randomisation process; (2) bias due to deviations from intended interventions; (3) bias due to missing outcome data; (4) bias in measurement of the outcome; and (5) bias in selection of the reported result. Overall study bias was scored using the algorithms provided in the RoB2.0 guidance documents. In brief, a study was judged as ‘low risk’ if the study scored low in all domains; ‘some concerns’ if the study was judged to raise some concerns in at least one domain but no high‐risk judgements; and ‘high risk’ if the study was judged to be either high in at least one domain or to have some concerns in multiple domains.

The studies assessed using ROBINS‐I and ROBINS‐E involved identifying preliminary confounders and assessing as either ‘low risk’, ‘moderate risk’, ‘serious/high risk’, or ‘critical risk’. For ROBINS‐I, scores were recorded for (1) bias due to confounding; (2) bias in selection of participants into the study; (3) bias in classification of interventions; (4) bias due to deviations from intended interventions; (5) bias due to missing data; (6) bias in measurement of outcomes; and (7) bias in selection of the reported result. For ROBINS‐E, scores were recorded for (1) bias due to confounding; (2) bias arising from measurement of the exposure; (3) bias in selection of participants into the study (or into the analysis); (4) bias due to post‐exposure interventions; (5) bias due to missing data; (6) bias in measurement of outcomes; and (7) bias in selection of the reported result. Overall study bias was judged ‘low risk’ if the study was judged as low for all domains; ‘moderate risk’ if judged to be low or moderate across the domains; ‘serious/high risk’ if judged serious in at least one domain; and ‘critical risk’ if judged to be critical in at least one domain (Sterne, Hernán, et al. 2016; Sterne, Higgins, et al. 2016).

### 2.6 Data Synthesis

Data are presented in a narrative synthesis in relation to the factors that contribute to the various outcome effects. This will help guide the future design and implementation of PA interventions on AMHUs. Key study characteristics and a summary of outcomes are presented in tables.

## 3 Results

Initial electronic database searches yielded 371 articles, with additional 10 articles identified from the grey literature. Updated searches yielded 169 papers from 2021 onwards; 129 of these had not been retrieved previously. The updated grey literature searches yielded a further 6 results.

**FIGURE 1:**
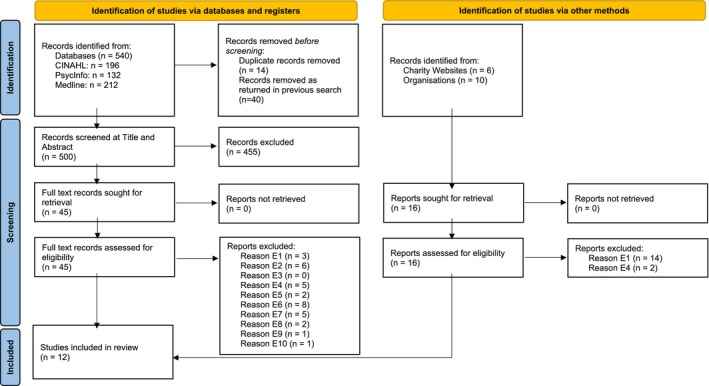
PRISMA flow diagram of search results and selection.

The PRISMA flow diagram can be seen in Figure 1, which provides further detail of the selection of studies and the selection criteria.

### 3.1 Study Characteristics

#### 3.1.1 Participants

Across the 12 eligible studies, there was a combined sample size of 560 (*n* = 12 studies), with the range in sample sizes from the smallest study having 16 (Curcic et al. 2017) participants to the largest having 80 participants (Sørensen et al. 2020). Participants were between 21 and 58 years of age. Only one study reported on ethnicity (Koch et al. 2019) with 100% (*n* = 32) of their sample reported as white Caucasian. Ten studies included patients with depressive disorder (Kim et al. 2014; Oertel‐Knöchel et al. 2014; Legrand and Neff 2016; Stanton et al. 2016; Koch et al. 2019; Haussleiter et al. 2020; Imboden et al. 2020; Sørensen et al. 2020; Polanco‐Zuleta et al. 2021; Torelly et al. 2022), four studies included patients with schizophrenia (Oertel‐Knöchel et al. 2014; Kim et al. 2014; Curcic et al. 2017; Areshtanab et al. 2020), three studies included patients with anxiety (Stanton et al. 2016; Koch et al. 2019; Sørensen et al. 2020), two studies included patients with bipolar disorder (Kim et al. 2014; Stanton et al. 2016), one study included patients with eating disorders (Koch et al. 2019), and one study included patients with psychosis (Sørensen et al. 2020).

#### 3.1.2 Setting

Eligible studies were conducted in Germany (Oertel‐Knöchel et al. 2014; Koch et al. 2019; Haussleiter et al. 2020), France (Legrand and Neff 2016), Iran (Areshtanab et al. 2020), Norway (Sørensen et al. 2020), Mexico (Polanco‐Zuleta et al. 2021), South Korea (Kim et al. 2014), Serbia (Curcic et al. 2017), Switzerland (Imboden et al. 2020), Brazil (Torelly et al. 2022), and Australia (Stanton et al. 2016). Description of the intervention settings is provided in Table 2.

**TABLE 2:** Study characteristics and intervention details for included articles.

Authors	Study design	Population		Physical activity intervention				Delivery			
		Description of setting	Participants	Activity	Type	Duration	Intensity	Delivered by whom	Individual/group	Additional Info	Control activity
Areshtanab et al. (2020)	Double‐blind RCT	Iran Inpatient psychiatric	Intervention (*n* = 34; Age M ± SD = 38.35 ± 6.64) Control (*n* = 34; Age = 37.26 ± 7.68)	Karvonen exercise program	Aerobic	3 pw × 20‐40 min; 8 weeks	65%–85% HR reserve	Nursing staff	Unclear	Outdoor	Unstructured outdoor time
Curcic et al. (2017)	RCT	Serbia inpatient clinic for mental disorders	Intervention (*n* = 40; 23 Male, 17 Female; Age = 39.95 ± 9.51) Control (*n* = 40; 19 Male, 21 Female; Age = 41.75 ± 9.45)	Individualised exercise programme	Aerobic	4 pw × 45 min; 12 weeks	65%–75% max HR	Fitness trainer	Unclear	Outdoor	Treatment as usual
Haussleiter et al. (2020)	RCT	German inpatient psychiatric centre	Intervention (*n* = 36; Age = 43.94 ± 13.24) Control (*n* = 40; Age = 46.43 ± 11.60)	Guided exercise therapy	Mixed	3 pw × 50 min; 6 weeks	NR	Exercise therapists	Group	—	Self‐organised activity
Imboden et al. (2020)	Double‐blind RCT	Swiss inpatient psychiatric centre	Intervention (*n* = 22; Age = 41.3 ± 9.2) Control (*n* = 20; Age = 38.3 ± 13.4)	Indoor cycling	Aerobic	3 pw × 45 min; 6 weeks	60%–75% max HR	External exercise therapists	Unclear	—	Supervised stretching
Kim et al. (2014)	RCT	South Korean inpatient psychiatry unit; closed ward	Intervention (*n* = 23; Age = 46.4 ± 2.1) Control (*n* = 19; Age = 50.8 ± 2.6)	Walking/running and other aerobic movements	Aerobic	3 pw × 60 min, 12 weeks	50% wk. 1–4; 60% wk. 5–8; 70% wk. 9–12. HR reserve	Instructor‐led	Unclear	—	Treatment as usual
Koch et al. (2019)	Pilot controlled study	German inpatient psychiatric hospitals.	Intervention (*n* = 16; Age = 31.63 ± 7.92), Control (*n* = 16; Age = 36.81 ± 13.23)	Flamenco therapy	Aerobic	1 × 60 min	NR	External trained music therapist	Group	—	Treatment as usual + talking therapy
Legrand and Neff (2016)	3‐group RCT	French inpatient mental health institution	Intervention (*n* = 14; Age = 45.3 ± 10.6), Placebo (*n* = 11; Age = 41.8 ± 13.2) Control (*n* = 10; Age = 49.1 ± 16.5)	Brisk walking or jogging	Aerobic	10 consecutive days × 30 min (exercise & stretching)	65%–75% max HR (exercise); 60 s hold/60 s rest (stretching)	Sports Scientist (1st author)	Mostly individual	Exercise group outdoors. Additional treatment = Stretching + medication Indoors	Treatment as usual
Oertel‐Knöchel et al. (2014)	3‐group RCT	German inpatient psychiatry department	Intervention (*n* = 16; Age = 41.13 ± 13.74), Placebo (*n* = 17; Age = 38.07 ± 12.01) Control (*n* = 18; Age = 40.41 ± 14.22) 8 patients with schizophrenia (SZ); 8 patients with MDD	Exercise group (boxing/circuits + cognitive training) OR Relaxation (+cognitive training	Aerobic	Intervention 3 pw × 75 min (cognitive training 30 min & exercise 45 min) 4 weeks; Placebo 3 pw × 75 min (cognitive training 30 min & relaxation 45 min); 4 weeks.	60%–70% max HR (exercise)	Trained physical exercise instructor; experience working with psychiatric patients	Group	Comparable psychosocial situations	Wait list
Polanco‐Zuleta et al. (2021)	Mixed method unblinded, non‐randomised quantitative study	Mexican psychiatric hospital of the Mexican Institute of Social Security	Groups combined (*n* = 27; Age = 29.89 ± 9.27); Intervention group *n* = 13; Control group *n* = 14)	Dance Program	Aerobic	6–8 days × 50 min	65%–89% max HR	Certified dance instructor	Group	21 primary caregivers also included (dance group *n* = 10; control *n* = 11)	Treatment as usual
Sørensen et al. (2020)	Qualitative	Inpatients psychiatric ward—Inpatient treatment for individuals with SMI. Normal duration of hospitalisation is around 8 weeks	Patients (*n* = 6; age range = 21 to 44; Female = 4), Staff (*n* = 6; age range = 28 to 43; Female = 3), 4 leaders (*n* = 4; age range 43 to 58; Female = 4). Staff & leaders = lead Psychologist, Specialist nurses and nurses	Physical activity with strength training	Mixed	2 pw × 60–75 min; 6.5 weeks.	NR	External physical activity instructors co‐delivered with local staff	Group	One session took place within the hospital and one took place in an external sports hall a 1 km walk away	NR
Stanton et al. (2016)	Cohort (with subgroup analysis)	Australia inpatient mental health ward; mean length of stay 22.4 days	Intervention (*n* = 40; Age = 45.1 ± 13.8)	Cycle/treadmill and resistance training	Mixed	2 pw × 40 min (20 min self‐selected intensity cycle/treadmill & 20 min resistance). For duration of stay (mean stay length = 22.4 days)	Self‐selected	Experienced exercise scientist	Group	—	NR
Torelly et al. (2022)	RCT crossover (3 groups)	Brazilian psychiatric inpatient unit	Total group (*n* = 33; Age = 37.03 ± 14.32)	Cycle and resistance training	Mixed	1 session × 38 min; 3 min of bi set resistance exercises (×5) with 3 min cycling in between	Moderate intensity; Borg RPE 12–14	NR	Unclear	—	Control = colouring/drawing activities

Abbreviations: HR, heart rate; NR, not reported; pw, per week; RCT, randomised control trial; RPE, rate of perceived exertion; SMI, severe mental illness.

#### 3.1.3 Diagnoses

Diagnostic tools used in establishing mental disorders and assessing symptom severity were the Diagnostic and Statistical Manual of Mental Disorders, version 4 (Oertel‐Knöchel et al. 2014; Kim et al. 2014; Legrand and Neff 2016; Haussleiter et al. 2020) and version 5 (Torelly et al. 2022), the International Classification of Diseases‐10 (ICD 10) codes (Curcic et al. 2017; Imboden et al. 2020; Sørensen et al. 2020), the Hamilton depression scale (Haussleiter et al. 2020; Imboden et al. 2020), psychiatrist diagnosed (Polanco‐Zuleta et al. 2021), the Beck Depression Inventory (Legrand and Neff 2016; Polanco‐Zuleta et al. 2021), and unvalidated self‐report (Stanton et al. 2016). Two studies did not report the method of diagnosis or symptom severity assessment (Koch et al. 2019; Areshtanab et al. 2020).

#### 3.1.4 Interventions

Across the 12 studies, six described group‐based exercise or physical activity (Oertel‐Knöchel et al. 2014; Stanton et al. 2016; Koch et al. 2019; Haussleiter et al. 2020; Sørensen et al. 2020; Polanco‐Zuleta et al. 2021), one described exercise or physical activity that was delivered on an individual basis (Legrand and Neff 2016), and five either did not report details on group size or it was unclear from the protocol description (Kim et al. 2014; Curcic et al. 2017; Areshtanab et al. 2020; Imboden et al. 2020; Torelly et al. 2022). Three studies reported that intervention delivery took place outdoors (Legrand and Neff 2016; Curcic et al. 2017; Areshtanab et al. 2020), two reported indoor sessions (Stanton et al. 2016; Koch et al. 2019), and seven either did not report the location or it was not clear from the setting description (Kim et al. 2014; Oertel‐Knöchel et al. 2014; Haussleiter et al. 2020; Imboden et al. 2020; Sørensen et al. 2020; Polanco‐Zuleta et al. 2021; Torelly et al. 2022).

All 12 studies included a physical activity component within their intervention and reported physical or psychological variables as their primary outputs (necessary to be eligible for this study). The type of physical activity interventions was traditional aerobic conditioning (i.e., fitness classes; Kim et al. 2014; Oertel‐Knöchel et al. 2014; Legrand and Neff 2016; Stanton et al. 2016; Curcic et al. 2017; Areshtanab et al. 2020; Haussleiter et al. 2020; Imboden et al. 2020), dance (Koch et al. 2019; Polanco‐Zuleta et al. 2021), or physical activity with strength training (Sørensen et al. 2020; Torelly et al. 2022).

### 3.2 Outcome Measures

Seven of the 12 studies reported improvements in mental health outcomes in favour of the intervention group (Oertel‐Knöchel et al. 2014; Legrand and Neff 2016; Koch et al. 2019; Areshtanab et al. 2020; Haussleiter et al. 2020; Polanco‐Zuleta et al. 2021; Torelly et al. 2022). Two studies reported improvements in physical health outcomes (Kim et al. 2014; Curcic et al. 2017). Two studies reported no differences between intervention and control groups for either mental health or physical health outcomes (Stanton et al. 2016; Imboden et al. 2020).

Physical and mental health outcomes from included studies are presented in Table 3. Whilst we aimed to explore the impact of physical activity interventions on system‐level outcomes including ward atmosphere and incidents of aggression or self‐harm, none of the included studies reported data on these outcomes. There was one qualitative study included in this review, and the authors reported positive experiences attributed to physical activity on peer and staff relationships from the perception of both patients and staff (Sørensen et al. 2020). In addition, patients from the same study described the physical activity intervention as having a positive impact on them physically, mentally, and socially (Sørensen et al. 2020). Staff and leaders reported experiencing conflict due to the additional demands on staff time and resources (e.g., equipment, facilities, and finances) to deliver a physical activity intervention, despite its benefits. They also emphasised the importance of utilising physical activity professionals (i.e., fitness instructors) suggesting that they do not necessarily see physical activity intervention delivery as part of their role (Sørensen et al. 2020).

**TABLE 3:** Quantitative outcome effects for the primary treatment arm in comparison to the control group and secondary treatment/placebo.

Authors	Primary treatment arm	Psychological outcome measures	Physical outcome measures	Key findings	Comparison between Primary intervention and control group^a^	Comparison between primary intervention and additional arm
Areshtanab et al. (2020)	Karvonen Exercise Programme	SQLS	NR	Sig. improvements in three SQLS domains for intervention (psychosocial, motivation and energy and side effects)	SQLS ↑	NR
Curcic et al. (2017)	Individualised exercise programme	PANSS	VO2max	Sig. increase in VO2max for intervention group. Sig. improvements in total PANSS score	VO2max ↑ PANSS overall ↑ PANSS positive ↔ PANSS negative ↔ PANSS general psychopathology ↔	NR
Haussleiter et al. (2020)	Guided exercise therapy	HAMD;PSP; WHO‐5; WSBB; FKB‐20, BFS Baseline; day 21 and day 43, FAHW baseline and 6 weeks	Timed Up & Go Test, Sit & Reach, Unipedal Stance Test	Improvements in overall HAMD. GET superior to SOA. No sig. difference between groups for WHO‐5. GET Sig. superior to SOA for reduction in subscales of HAMD (Early & Middle insomnia, psychomotor retardation and anxiety)	HAMD total ↑ (partial *η* ^2^ = 0.12) PSP total ↔ (partial *η* ^2^ = 0.01) WHO‐5 ↔ (partial *η* ^2^ = 0.06) WSBB (NR) FKB‐20 ‘body vitality’ ↑ (t × g) BFS ‘tension’ ↑ (t × g) FAHW ‘physical ill‐being’ ↑ (t × g) Timed Up & Go ↔ Sit & Reach ↔ Unipedal Stance Test ↔	NR
Imboden et al. (2020)	Indoor cycling	HRDS17; BDI (baseline, wk. 1, 2, 6 & 6 month); MTQ18; PDSQ (baseline, wk. 6 & 6 month) IPAQ (baseline & 6 month)	Queens College Step Test (VO2max), body weight, BMI, blood pressure and heart rate	HRDS7 & BDI sig. decreased after 6 weeks but no sig. diffs between groups. Long term no further improvements. Increase in PA regardless of group (non‐Sig.)	Short term effects (6 weeks post intervention) BDI ↔ (t × g) (*η* ^2^ = 0.00) PDSQ ↔ (t × g) (*η* ^2^ = 0.00) HDRS17 ↔ (t × g) (*η* ^2^ = 0.00) MTQ18 ↔ (t × g) (*η* ^2^ = 0.04) BMI ↔ (t × g) (*η* ^2^ = 0.02) Systolic BP ↔ (t × g) (*η* ^2^ = 0.00) Diastolic BP ↔ (t × g) (*η* ^2^ = 0.00) Resting HR ↔ (t × g) (*η* ^2^ = 0.03) IPAQ ↔ (t × g) VO2max ↔ (t × g) (*η* ^2^ = 0.00)	NR
Kim et al. (2014)	Walking/running & other Aerobic movements	NR	Body weight, BMI, lean body mass, body fat %, blood pressure, resting HR, thigh circumference, waist circumference, grip strength, leg strength, YMCA bench step test, jump, reach	Decreases in weight & BMI, but non‐Sig between groups. Intervention group Sig improvements in leg muscle strength, cardiorespiratory and jump	Body weight ↔ (t × g) BMI ↔ (t × g) Lean body mass ↔ (t × g) Body fat % ↔ (t × g) Systolic BP ↓ (t × g) Diastolic BP ↔ (t × g) Resting HR ↔ (t × g) Thigh circumference ↔ (t × g) Waist circumference ↔ (t × g) Grip strength ↔ (t × g) Leg strength ↑ (t × g) YMCA bench step ↑ (t × g) Jump ↑ (t × g) Reach ↔ (t × g)	NR
Koch et al. (2019)	Flamenco Therapy	HIS; BSE; EIS	Perceived levels of fitness, physical pain and general health	Statistically significant improvements psychological well‐being score, and perceived level of health and pain in experimental group	HIS positive ↔ (t × g) (eta^2^ = 0.02) HIS negative ↑ (t × g) (eta^2^ = 0.14) BSE ↔ (t × g) (eta^2^ = 0.02) EIS ↔ (t × g) (eta^2^ = 0.10) Perceived fitness ↔ (t × g) (eta^2^ = 0.01) Pain ↑ (t × g) (eta^2^ = 0.12) Health ↑ (t × g) (eta^2^ = 0.38)	NR
Legrand and Neff (2016)	Brisk walking or jogging	BDI‐II	NR	Sig. improvement in BDI score in aerobic group pre to post intervention compared to control. Sig. improvement in BDI score in placebo (stretching) pre to post. No sig difference between aerobic and stretching groups, no sig differences between stretching and control	BDI‐II ↑ (t × g) (Cohen's *d* = −1.39)	BDI‐II ↔ (t × g) (Cohen's *d* = −1.06)
Oertel‐Knöchel et al. (2014)	Boxing/circuits	STAI, PSK of SF12, BDI‐II (MDD patients only), PANSS (SZ patients only); RHS (SZ patients only)	NR	Reduction in severity of illness; strongest effects in exercise group when compared to control and relaxation groups	^b^ MDD patients STAI ↑ (Cohen's *d* = 0.7) BDI‐II ↑ (Cohen's *d* = 0.91) ^b^ SZ patients PANSS pos ↔ (Cohen's *d* = 0.11) PANSS neg ↑ (Cohen's *d* = 0.24) RHS ↔ (Cohen's *d* = 0.11)	^b^ MDD patients STAI ↑ (Cohen's *d* = 0.95) BDI‐II ↑ (Cohen's *d* = 0.58) ^b^ SZ patients PANSS pos ↔ (Cohen's *d* = 0.23) PANSS neg ↑ (Cohen's *d* = 0.24) RHS ↔ (Cohen's *d* = 0.12)
Polanco‐Zuleta et al. (2021)	Dance program	GSE; BDI; semi‐structured interview for primary caregivers	HRV HR	Sig. increase in self‐efficacy in dance group. Sig. decrease in depression in both groups but larger effect in dance group	GSE ↑ (t × g) (ƞp^2^ = 0.42) BDI ↑ (t × g) (ƞp^2^ = 0.44) HRV ↔ (t × g) (ƞp^2 =^ 0.03) HR ↑ (t × g) (ƞp^2^ = 0.15)	NR
Stanton et al. (2016)	Cycle/treadmill & resistance training	FS; FAS	NR	Compared with pre‐exercise scores, affective valance (FS) scores sig. higher after exercise for people with bipolar and depressive disorders but not those with anxiety. No sig. pre‐post difference in FAS found for any diagnostic category.	FS ↔ FAS ↔	NR
Torelly et al. (2022)	Cycling & resistance training	STAI; PANAS; VA‐WB	Brain Derived Neurotrophic Factor	All interventions increased well‐being and positive affect and reduced anxiety and negative affect. EX had larger effects on increasing well‐being. MB had larger effects reducing negative affect	^b^ Exercise group STAI ↑ (Cohen's *d* = −0.4) PANAS pos ↑ (Cohen's *d* = 0.5) PANAS neg ↑ (Cohen's *d* = −0.2) VA‐WB ↑ (Cohen's *d* = 0.8) BDNF ↑ (Cohen's *d* = 0.3) Control group STAI ↑ (Cohen's *d* = −0.3) PANAS pos ↑ (Cohen's *d* = 0.3) PANAS neg ↔ (Cohen's *d* = −0.1) VA‐WB ↑ (Cohen's *d* = 0.4) BDNF ↑ (Cohen's *d* = 0.5)	^b^ MindBody group STAI ↑ (Cohen's *d* = −0.5) PANAS pos ↑ (Cohen's *d* = 0.5) PANAS neg ↑ (Cohen's *d* = −0.4) VA‐WB ↑ (Cohen's *d* = 0.5) BDNF ↑ (Cohen's *d* = 0.3)

*Note:* t × g = time × group interaction; ↑ = Significant improvement in outcomes; ↔ = no significant change in outcomes.

Abbreviations: BACS SC, Brief Assessment of cognition in Schizophrenia; BDI, Beck Depression Inventory; BFS, Befindlichkeitsskala; BMI, Body Mass Index; BSE, Body Self‐Efficacy Scale; BVMT‐R, Brief Visuospatial Memory Test‐Revised; CTT, Colon Transit Time; EIS, Embodied Intersubjectivity Scale; EX, Exercise; FAHW, German Questionnaire on General Habitual Subjective Well‐Being; FAS, Felt Arousal Scale; FKB‐20, Fragebogen zum Körperbild‐20; FS, Feeling Scale; GSE, General Self‐Efficacy Scale; HAMD, Hamilton Depression Rating Scale; HDRS17, Hamilton Depression Scale 17; HIS, Heidelberg State Inventory; HR, Heart Rate; HVLT‐R, Hopkins Verbal Learning Test‐Revised; IPAQ, International Physical Activity Questionnaire; LNS, Letter‐Number Span; MB, Mind–Body; MDD, Major Depressive Disorder; MTQ, Mental Toughness Questionnaire; PA, Physical Activity; PANAS, Positive and Negative Affect Scale; PANSS, Positive and Negative Symptoms Scale; PDSQ, Physical Self‐Description Questionnaire; PSK of SF 12, psychic subscale of SF‐12; PSP, Personal and Social Performance Scale; QOLS, Quality of Life Scale; RHS, Revised Hallucination Scale; SOA, Self‐Organised Activity; STAI, State–Trait‐Anxiety Inventory; SZ, Schizophrenic; TAP, Testbatterie zur Aufmerksamkeitprüfung; TMT, Trail Making Test; VA‐WB, Visual Analog Well‐Being Scale; WHO‐5, WHO Well‐Being Index; WMS, Wechsler Memory Test; WSBB, Weinsberger Skalen zur Bewegungsbeobachtung.

^a^ Between group effect reported unless otherwise indicated.

^b^ Within group (baseline to post change) effect sizes presented as effect size for between group comparison not available.

### 3.3 Effectiveness of Physical Activity Intervention

Studies that included a control group reported treatment as usual, including pharmacological treatment with no exercise (Kim et al. 2014; Legrand and Neff 2016; Curcic et al. 2017; Koch et al. 2019; Polanco‐Zuleta et al. 2021), active stretching (Imboden et al. 2020), alternative active exercise (Areshtanab et al. 2020; Haussleiter et al. 2020), talking therapy (Koch et al. 2019), colouring activities (Torelly et al. 2022), and wait list (Oertel‐Knöchel et al. 2014).

In addition, three studies reported additional treatment arms in addition to their control group, including stretching plus medication (Legrand and Neff 2016), relaxation plus cognitive training (Oertel‐Knöchel et al. 2014), and mind–body practices (Torelly et al. 2022). Two studies did not report specific treatment as usual, but their control group was identified as having an alternative physical activity treatment (Areshtanab et al. 2020; Haussleiter et al. 2020). Comparison between primary treatment and additional treatment arms is presented in Table 3.

Studies that reported positive effects included group exercise (Kim et al. 2014; Oertel‐Knöchel et al. 2014; Koch et al. 2019; Haussleiter et al. 2020; Polanco‐Zuleta et al. 2021), guided/instructed exercise (Oertel‐Knöchel et al. 2014; Curcic et al. 2017; Koch et al. 2019; Areshtanab et al. 2020; Haussleiter et al. 2020; Polanco‐Zuleta et al. 2021), outdoor exercise (Legrand and Neff 2016; Curcic et al. 2017; Areshtanab et al. 2020), and included caregivers in the activity (Oertel‐Knöchel et al. 2014). Studies that reported no notable effects compared to controls included self‐selected exercise intensity, indoors on a cycle ergometer (Stanton et al. 2016; Imboden et al. 2020). However, the lack of comparative analysis across interventions in a number of studies and the substantial heterogeneity in the design and implementation of interventions in this review meant that it was not possible to make firm inferences on specific intervention components that contributed to more positive outcomes.

### 3.4 Risk of Bias

**FIGURE 2:**
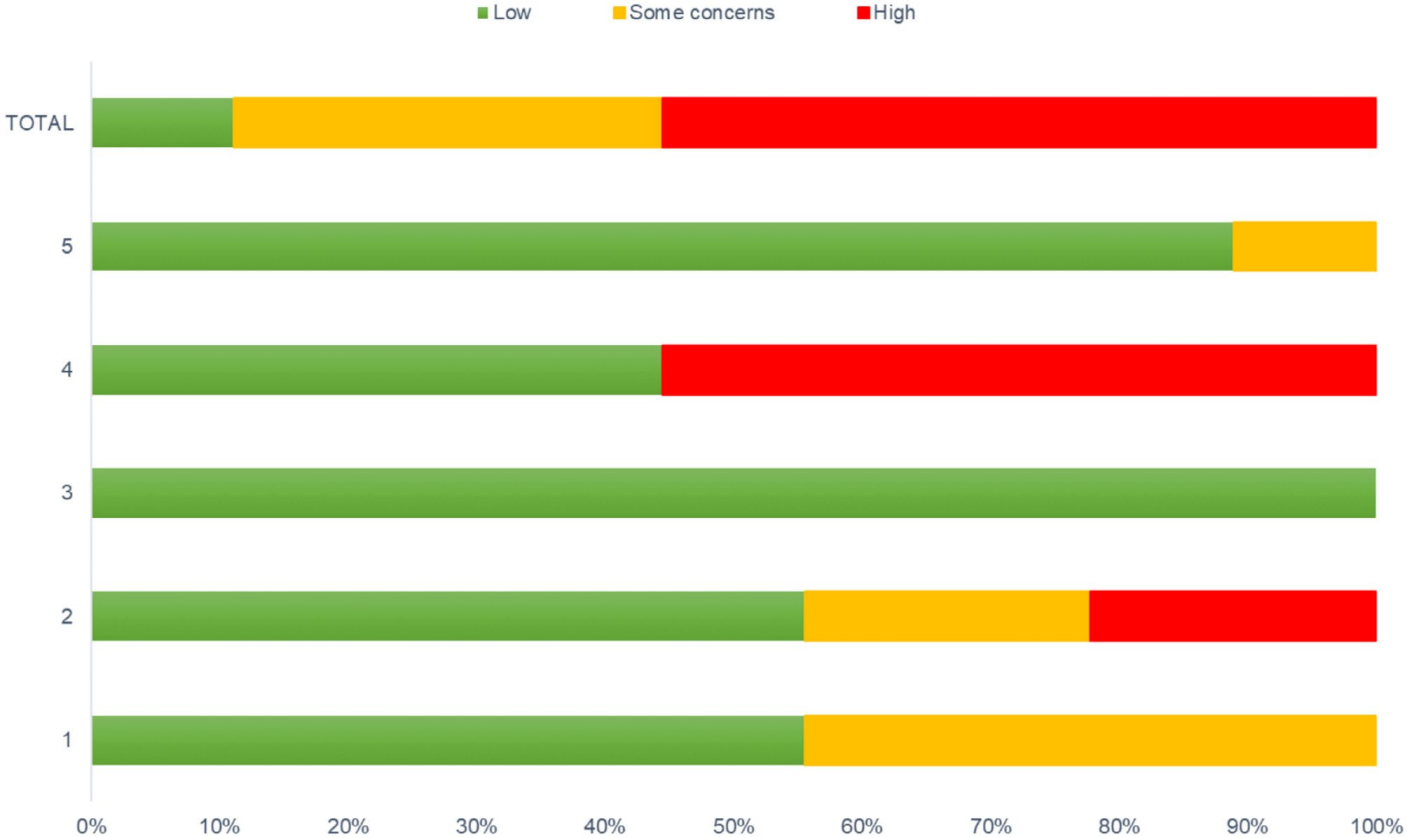
Summary of the risk of bias assessment for randomised control trials. 1 = bias arising from the randomisation process; 2 = bias due to deviations from intended interventions; 3 = bias due to missing outcome data; 4 = bias in measurement of the outcome; 5 = bias in selection of the reported result.

Summaries of the risk of bias assessment for the nine included RCT studies are presented in Figure 2. The risk of bias for the non‐RCT studies is presented in Table 4. A qualitative study was not assessed, and therefore, the information that follows is exclusive of Sørensen et al. (2020). Only one study was recorded as having a low risk of bias across all domains (Imboden et al. 2020) with 56% of studies scored as high risk overall. Overall, when scoring and extracting data related to risk‐of‐bias and quality appraisal, there were consistent shortcomings. For example, areas that contributed most to a high risk of bias were possible deviations from intended interventions (Koch et al. 2019; Polanco‐Zuleta et al. 2021), bias in measurement of the outcome (Kim et al. 2014; Legrand and Neff 2016; Stanton et al. 2016; Curcic et al. 2017; Koch et al. 2019), and inappropriate analysis techniques (Torelly et al. 2022). Similarly, appraisal using the MMAT highlighted that there was a consistent underreporting of details relating to blinding of outcome assessors and the adherence to assigned interventions. Further, sufficient information on attrition and adherence across the 11 studies assessed made it hard to identify if the data analysed was conducted on a complete data set or how instances of missing data were dealt with. Where this data was reported, the methods applied (i.e., Intention‐to‐treat; LOCF) have been previously criticised (Andrade 2022). Where this is paired with underpowered samples (Legrand and Neff 2016; Koch et al. 2019; Imboden et al. 2020; Polanco‐Zuleta et al. 2021; Torelly et al. 2022) and inappropriate analysis techniques (Torelly et al. 2022), cautious interpretation of significant time by group interactions is recommended.

**TABLE 4:** Risk of bias assessment for non‐randomised studies of interventions (ROBINS‐I) and exposures (ROBINS‐E).

**ROBINS‐E**	Bias arising due to:							
	Confounding	Measurement of the exposure	Selection of participants into the study	Post‐exposure interventions	Missing data	Measurement of outcomes	Selection of the reported result	Total
Stanton et al. (2016)	**High**	**Low**	**Low**	**Low**	**Low**	**High**	**Low**	**High**
**ROBINS‐I**	Bias arising due to:							
	Confounding	Selection of participants into the study	Classification of interventions	Deviations from intended interventions	Missing data	Measurement of outcomes	Selection of the reported result	Total
Polanco‐Zuleta et al. (2021)	**Low**	**Serious**	**Low**	**Critical**	**No Info**	**Low**	**Moderate**	**Critical**

## 4 Discussion

Individuals with serious mental illness are dying 10–20 years earlier than the general population (Plana‐Ripoll et al. 2020). Seventy percent of premature mortality is linked with physical health conditions which are modifiable with physical activity, diet, and smoking cessation (Walker et al. 2023). This review aimed to explore the factors that are important to the successful design and implementation of physical activity interventions with specific reference to AMHU's. We identified 12 physical activity intervention studies that were eligible for inclusion in the review. The results highlighted that physical activity interventions on AMHU's have the potential to contribute to improvements in mental and physical wellbeing beyond that experienced from usual treatment practices (e.g., medication). Ashdown‐Franks et al. (2020) conducted an umbrella review of 27 systematic reviews and concluded similar findings, that physical activity interventions can be an effective treatment across a range of mental health conditions. However, implementation methods and successful outcomes of any physical activity intervention will vary depending on the environment in which it is delivered (Ashdown‐Franks et al. 2020), and it is not known if these positive outcomes translate to interventions delivered on AMHU's.

Due to heterogeneity between studies, in particular the large variation between intervention characteristics, and the high risk of bias identified, it was not possible to complete a meta‐analysis of the studies included in this review. Heterogeneity and quality concerns relating to physical activity intervention studies focused on individuals with mental health conditions have been previously discussed (Peckham et al. 2023). The high risk of bias for studies in this review was introduced largely due to the inability to blind outcome assessors when using patient‐reported outcome measures (PROMs) in the assessment of mental health outcomes. Whilst these self‐report measures make it hard to establish an objective change in outcomes correlated to physical activity on the markers of psychological wellbeing, the benefits to wellbeing through increased attention and focus the patient receives during the intervention itself, and through the consciousness‐raising of self‐report measures may be perceived as positive outcomes in itself.

Across all studies, there was a lack of clarity when reporting on specific details related to the intervention, such as an accurate description of the setting, and in the description of the allocation of participants, intervention implementation, reporting on the outcome measure methodology, and information on possible deviations from intended interventions. These key omissions are critical when trying to establish information about how and why the implementation of the intervention was successful or not, making it difficult for future intervention development and implementation in similar settings (Skivington et al. 2021). For example, in this review, only one study reported the ethnicity of the participants (Koch et al. 2019). To be able to identify inequalities in access to services within acute mental health care, future investigators should report more detail in relation to patient demographics, including ethnicity. Therefore, future intervention studies should consider a more extensive use of the TIDieR framework (Hoffmann et al. 2014) to improve the completeness and replicability of interventions.

To establish whether the mental and physical health improvements highlighted in nine of the 12 included studies are linked to changes in physical activity behaviour, it is important to track any change in physical activity during and post intervention. However, only two of the 12 studies collected and reported data on physical activity from baseline to post‐intervention (Haussleiter et al. 2020; Imboden et al. 2020). Haussleiter et al. (2020) explored the effect of a combined guided exercise therapy (by certified therapists) plus self‐organised activities compared to self‐organised activity alone, on mental health outcomes in inpatients with major depressive disorder. Physical activity was tracked using fitness journals with patients asked to record the number of minutes spent in self‐organised activity each week. Imboden et al. (2020) compared group aerobic exercise at 60%–75% age‐predicted HrMax compared to group stretching, on a variety of physical and mental health outcomes. Physical activity was tracked using the International Physical Activity Questionnaire (IPAQ) (Craig et al. 2003). Imboden et al. (2020) found no significant differences between groups for mental health outcomes, physical health outcomes, or IPAQ‐reported physical activity. In Haussleiter et al. (2020) the guided exercise group performed less overall activity over the weekly sessions but reported better total scores on the Hamilton Depression Scale. When taken together, the results of these two studies may infer that it is other aspects of the group exercise; for example, peer relationships, that might have had a mediating effect (Johns et al. 2019; Wainberg et al. 2020).

Whilst the findings of this review are similar to previous reviews on this topic (Ashdown‐Franks et al. 2020; Hassan et al. 2022; Peckham et al. 2023), this is the first review to attempt to consolidate the potential benefits of physical activity interventions in the unique context of AMHUs only. The specific environmental context is important when considering the likelihood of the intervention meeting the intended outcomes (Skivington et al. 2021); simply lifting interventions that have worked in other hospitals or even mental health settings (Hassan et al. 2022) risks missing the contextual nuances that may be a barrier or facilitator to successful intervention implementation (Potthoff et al. 2023).

This is especially important when considering the uniquely challenging nature of an AMHUs, where high levels of distress and relatively brief periods of stay (Gilburt and Mallorie 2024) are paired with limited resources (Raphael et al. 2021). Previous attempts at implementing a variety of interventions in an AMHU have identified issues relating to intervention fidelity (Bowers et al. 2015), staff capability (e.g., training) and opportunity (multi‐disciplinary teams and change culture) (Raphael et al. 2021). One qualitative study included in this review found that there were positive experiences attributed to physical activity on peer and staff relationships and that patients reported positive impacts (Sørensen et al. 2020). However, staff and leaders were conflicted due to additional demands on staff to deliver physical activities. Given the current crisis in shortages of mental health nursing (NHS Confederation 2023), it is critical that consideration for additional staff and resources is factored into future study design, or at the very least, it does not add additional strain on stretched and exhausted clinical teams.

This review provides the foundation on which to consider study designs for physical activity interventions in an AMHU. However, there are a number of limitations to note. First, the search was restricted to studies published post 2013 to coinciding with the introduction of ‘Parity of Esteem’ policy (Royal College of Psychiatrists 2013). Whilst we recognise that earlier intervention studies are available, limiting to post‐2013 allowed for the consideration of evidence following this policy change. Studies were included regardless of geographical location, but we decided to restrict searches by the UK policy date as it preceded other international mental health policy (e.g., the EU commission approach to mental health).

Furthermore, the purpose of this review is to guide the future design and implementation of PA interventions; thus, the selection of papers in the last 10 years more accurately reflects the current landscape. Second, whilst we highlight the potential effectiveness of physical activity interventions on the mental and physical health of inpatients on AMHUs, the reporting quality and heterogeneity between included studies make it difficult to offer any conclusive evidence. This limitation further highlights a domain‐specific need for improvement in reporting the details related to the intervention.

A primary aim of this review was to establish the effectiveness of physical activity interventions on AMHUs. However, the lack of objective physical activity measurement made it hard to establish if the improvement in the mental health outcomes was indeed linked to any increase in physical activity. Future interventions should consider the inclusion of an objective measure of physical activity to complement the current assessment of physical and mental outcomes reported in these studies.

We also found no UK‐based studies that met the inclusion criteria for this review. This might be due to the challenges and resource pressures of working in an AMHU (Raphael et al. 2021) or more likely due to limited dissemination of local studies which use a service improvement methodology and which are largely unpublished, despite showing promising results. We recommend future localised initiatives access support to design and implement their studies using robust research methods and frameworks, which could encourage publication and wider dissemination of good practice in this highly complex environment.

## 5 Conclusions

Physical activity interventions on AMHU's have the potential to contribute to improvements in mental and physical wellbeing. However, the results from our study would suggest that further work is needed in the specific context of AMHU's regarding the development of physical activity interventions, using the MRC framework principles to gain a better understanding of the context and wider factors for intervention development and success (Skivington et al. 2021). This review provides the first step in this process and highlights the current evidence on the effectiveness of physical activity interventions in AMHUs on physical and mental health outcomes. The limited number of studies in this review and the heterogeneity between these studies highlight the need for further exploration of the setting, including associated complexities, along with patient and staff involvement in intervention development, implementation, and evaluation.

## 6 Relevance to Clinical Practice

The seventy‐five percent of studies reviewed reported positive physical and/or mental health outcomes following a physical activity intervention, suggesting that physical activity interventions in this setting can act as an adjunct treatment. To improve the quality of future study design, there is a need to increase the quality in the reporting of the complexities of the setting and intervention implementation. We recommend the involvement of both patients and staff as part of future intervention development, implementation, and evaluations in this unique setting.

## Author Contributions

Michael Graham: project administration, conceptualization, data curation, investigation, formal analysis, methodology, visualisation, writing – original draft, writing – review and editing. Philip Hodgson: conceptualization, data curation, investigation, methodology, writing – review and editing. Laura Fleming: conceptualization, data curation, visualisation, writing – review and editing. Alison Innerd: funding acquisition, data curation, methodology, writing – review and editing. Nicola Clibbens: conceptualization, methodology, investigation, writing – review and editing, supervision. Wendy Hope: investigation, resources, writing – review and editing. Luke Aston: data curation, visualisation, writing – original draft, writing – review and editing. Michelle Glascott: funding acquisition, conceptualization, visualisation, writing – original draft, writing – review and editing, supervision.

## Conflicts of Interest

The authors declare no conflicts of interest.

## Supporting information


Data S1.

## Data Availability

The data that supports the findings of this study are available within the manuscript text, tables and figures.
