# Supplementary material for: Effectiveness of Physical Activity Interventions on Acute Inpatient Mental Health Units on Health Outcomes: A Systematic Review
Source: Int J Ment Health Nurs. 2025 Feb 23;34(1):e70017. doi: 10.1111/inm.70017 (PMC11848114; doi:10.1111/inm.70017)
Supplement: Supplementary file 1 — Data S1. [file INM-34-0-s001.docx]

**CINAHL**

| **#** | **Query** |  |
| --- | --- | --- |
| S20 | S17 AND S18 AND S19 |  |
| S19 | S13 OR S14 OR S15 OR S16 |  |
| S18 | S8 OR S9 OR S10 OR S11 OR S12 |  |
| S17 | S1 OR S2 OR S3 OR S4 OR S5 OR S7 |  |
| S16 | TI,AB "ward-based" OR "acute ward*" OR "acute unit*" |  |
| S15 | TI,AB hospitalised or hospitalized |  |
| S14 | (psychiatric or mental) N2 (inpatient* or in-patient*) |  |
| S13 | (MH "Inpatients") |  |
| S12 | psych* N1 (illness* or disorder*) |  |
| S11 | mental N1 (illness* or disorder*) |  |
| S10 | (MH "Panic Disorder") OR (MH "Depression+") OR (MH "Stress Disorders, Post-Traumatic+") OR (MH "Somatoform Disorders") OR (MH "Psychotic Disorders+") OR (MH "Substance Use Disorders+") OR (MH "Adjustment Disorders") OR (MH "Organic Mental Disorders") |  |
| S9 | (MH "Mental Disorders") OR (MH "Personality Disorders") OR (MH "Anxiety Disorders") OR (MH "Factitious Disorders+") OR (MH "Dissociative Disorders") OR (MH "Affective Disorders") OR (MH "Obsessive-Compulsive Disorder") OR (MH "Phobic Disorders") OR (MH "Eating Disorders+") OR (MH "Sleep Disorders+") |  |
| S8 | (MM "Mental Health") |  |
| S7 | (MM "Exercise") OR (MM "Aerobic Exercises+") OR (MM "Callisthenics") OR (MM "Group Exercise") OR (MM "Cycling") |  |
| S6 | (MM "Physical Activity") |  |
| S5 | MM ("sports") |  |
| S4 | TI,AB "physical rehabilitat*" OR "physical exertion" |  |
| S3 | MM ("physical fitness") |  |
| S2 | TI,AB e-exercise or workout or "couch to 5K" or dance or dancing |  |
| S1 | physical N3 (activ* or fit*) |  |

**EMBASE**

1 *mental disease/ or exp *addiction/ or exp *adjustment disorder/ or anxiety disorder/ or exp *autism/ or exp *dissociative disorder/ or exp *mood disorder/ or *neurosis/ or exp *personality disorder/ or exp *psychosis/ or exp *schizophrenia spectrum disorder/ or exp *eating disorder/ or *obsessive compulsive disorder/

2 (mental adj1 (illness* or disorder* or health)).ti,ab.

3 ((psychiatric or mental) adj1 (inpatient* or in-patient*)).ti,ab.

4 (psych* adj1 (illness* or disorder*)).ti,ab.

5 *hospital patient/

6 (hospitalised or hospitalized).ti,ab.

7 *mental patient/

8 (ward-based or "acute ward" or "acute unit").ti,ab.

9 *physical activity/

10 *sport/

11 *exercise/ or *aerobic exercise/ or *calisthenics/ or *exergaming/ or *cycling/

12 ("physical rehabilitat*" or "physical exertion").ab,ti.

13 (e-exercise or workout or "couch to 5K" or dance or dancing).ab,ti.

**PsycInfo**

| Set# | Searched for |
| --- | --- |
| S2 | MJMAINSUBJECT.EXACT("Physical Activity") OR MJMAINSUBJECT.EXACT("Exercise") OR MJMAINSUBJECT.EXACT("Sports") |
| S4 | (physical NEAR/3 (activ* OR fit*)) OR TIAB(e-exercise or workout or "couch to 5K" or dance or dancing or aerobic* or calisthenic* or cycling) OR TIAB("physical rehabilitat*" OR "physical exertion") |
| S5 | MJMAINSUBJECT.EXACT("Aerobic Exercise") OR MJMAINSUBJECT.EXACT("Cycling") |
| S6 | MJMAINSUBJECT.EXACT("Mental Health") OR MJMAINSUBJECT.EXACT("Mental Disorders") |
| S7 | MJMAINSUBJECT.EXACT.EXPLODE("Dissociative Disorders") OR MJMAINSUBJECT.EXACT.EXPLODE("Personality Disorders") OR MJMAINSUBJECT.EXACT.EXPLODE("Anxiety Disorders") OR MJMAINSUBJECT.EXACT.EXPLODE("Affective Disorders") OR MJMAINSUBJECT.EXACT.EXPLODE("Obsessive Compulsive Disorder") OR MJMAINSUBJECT.EXACT.EXPLODE("Eating Disorders") OR MJMAINSUBJECT.EXACT("Sleep Wake Disorders") OR MJMAINSUBJECT.EXACT.EXPLODE("Somatoform Disorders") |
| S8 | (MJMAINSUBJECT.EXACT.EXPLODE("Psychosis") or TIAB("psychotic disorder*")) OR MJMAINSUBJECT.EXACT.EXPLODE("Schizophrenia") |
| S9 | (mental N/1 (illness* or disorder*)) OR (psych* N/1 (illness* or disorder*)) |
| S11 | MJMAINSUBJECT.EXACT("Psychiatric Patients") AND MJMAINSUBJECT.EXACT("Hospitalized Patients") |
| S13 | TIAB("ward-based" OR "acute ward*" OR "acute unit* or "acute admission*"") |
| S14 | (psychiatric or mental) N/2 (inpatient* or in-patient*) |
| S15 | TIAB(hospitalised or hospitalized) |
| S16 | S2 or s4 or s5 |
| S17 | s6 or s7 or s8 or s9 |
| S18 | s11 or s13 or s14 or s15 |
| S19 | s16 and s17 and s18 |
| S23 | (s16 and s17 and s18) AND (la.exact("ENG") AND su.exact("Middle Age (40-64 yrs)" OR "Young Adulthood (18-29 yrs)" OR "Thirties (30-39 yrs)") AND pd(20090101-20221231) AND PEER(yes)) |
